# Observation of in-plane exciton–polaritons in monolayer WSe_2_ driven by plasmonic nanofingers

**DOI:** 10.1515/nanoph-2022-0201

**Published:** 2022-05-16

**Authors:** Guangxu Su, Anyuan Gao, Bo Peng, Junzheng Hu, Yi Zhang, Fanxin Liu, Hao Zhang, Peng Zhan, Wei Wu

**Affiliations:** National Laboratory of Solid State Microstructures, Collaborative Innovation Center of Advanced Microstructures and School of Physics, Nanjing University, Nanjing 210093, China; Department of Applied Physics, Zhejiang University of Technology, Hangzhou 310023, China; Department of Optical Science and Engineering, Fudan University, Shanghai 200433, China; College of Energy and Electrical Engineering, Hohai University, Nanjing, 210098, China; Department of Electrical Engineering-Electrophysics, University of Southern California, Los Angeles, CA 90089, USA

**Keywords:** collapsed nanofingers, exciton–polaritons, in-plane strong coupling, monolayer WSe_2_, photoluminescence

## Abstract

The transition metal dichalcogenides (TMDs) have drawn great research attention, motivated by the derived remarkable optoelectronic properties and the potentials for high-efficient excitonic devices. The plasmonic nanocavity, integrating deep-sub wavelength confinement of optical mode with dramatic localized field enhancement, provides a practical platform to manipulate light–matter interaction. In order to obtain strong exciton–plasmon coupling effects, it’s crucial to match the vibration direction of exciton to the available strong localized in-plane electric field. Herein, we demonstrate the coupling effect of in-plane exciton in monolayer tungsten diselenide (WSe_2_) to deterministic gap-plasmon field which is produced by nanometrically gapped collapsed nanofingers. The gap-plasmon field which is completely parallel to the in-plane excitons in WSe_2_ will drive a strong exciton–plasmon coupling at room temperature. More interestingly, it is experimentally observed that the luminescence of exciton–polariton cannot be influenced by the temperature in the range from 77 K to 300 K due to the presence of nanofingers. According to the theoretical analysis results, we attribute this finding to the dielectric screening effect arising from the extremely strong localized electric field of plasmonic nanofingers. This work proposes a feasible way to harness and manipulate the exciton of low-dimensional semiconductor, which might be potential for quantum optoelectronics.

## 1 Introduction

Recently, the monolayer transition metal dichalcogenides (TMDs) have drawn great attentions due to their unique optical and electronic properties, such as the direct bandgap with tunable exciton binding energies in wide region from visible to near infrared [1], [2], [3], and are promising candidates for ultracompact and flexible exciton-based on-chip devices [4], [5], [6]. The TMDs have emerged as an important platform for exploring the coupling between excitons and photons because the spatial distributions and orientations of the excitons are controllable in compared with J-aggregates [7], [8], [9], quantum dots [10], or molecules [11]. In this coupling system, the exciton–polaritons are half-light half-matter quasi-particle and can bridge photonic and electronic systems. The manipulation of light–matter interaction can induce a definite Rabi splitting in the dispersion curves and can be applied to single-photon source and low-threshold lasers [12]. The research work on strong coupling of exciton–polaritons in TMDs have been widely carried out at real and momentum space, using plasmonic nanocavity [13], [14], [15], [16], [17], plasmonic lattice [18, 19], photonic crystals [20], [21], [22], and other nanophotonic structures [23, 24]. Among these systems, the gap plasmon is preferred due to the ability to produce a dramatic localized field enhancement with nanoscale mode volume, which can enhance the coupling strength [25]. In addition, matching the vibration directions of excitons and plasmonic electromagnetic field is also essential to the coupling.

**Figure 1:**
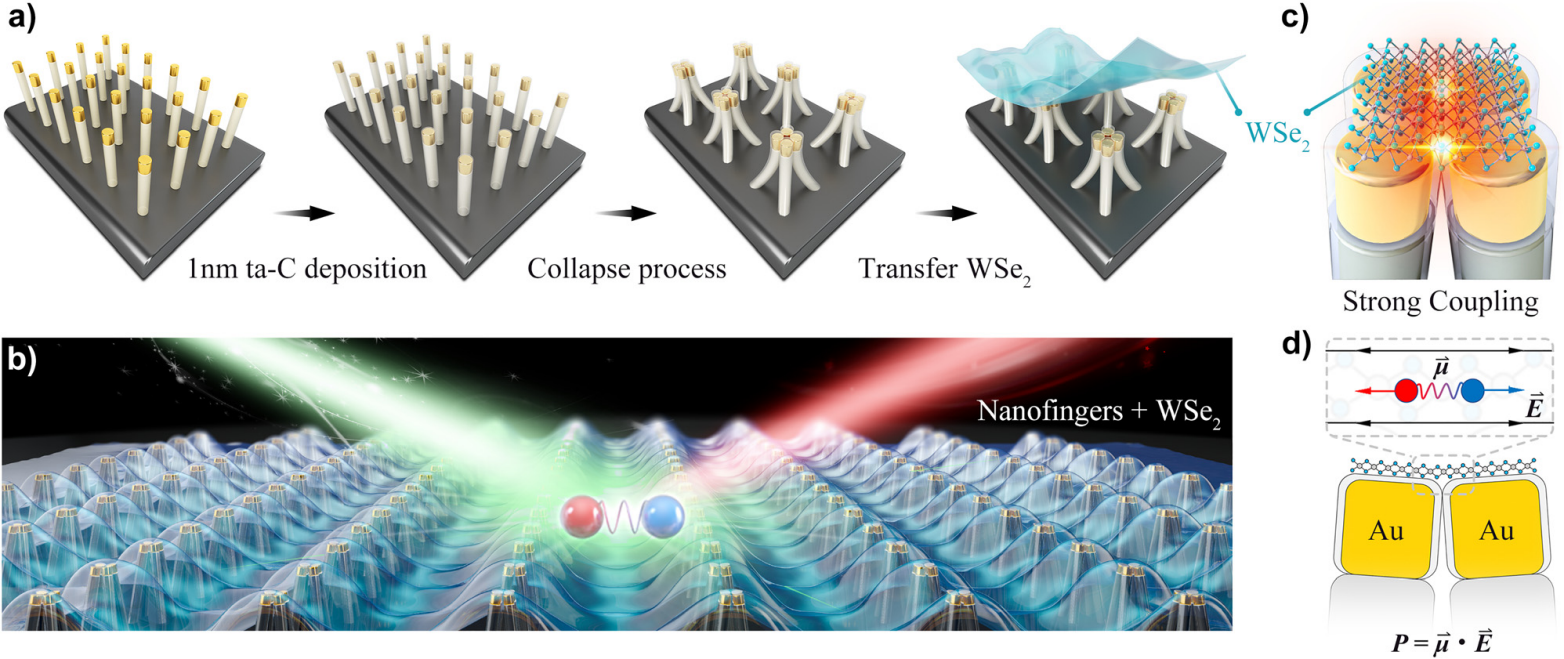
In-plane exciton–plasmon polaritons in monolayer WSe_2_ driven by plasmonic nanofingers. (a) Schematic of fabrication process of monolayer WSe_2_ on plasmonic nanofinger arrays. (b) Strong coupling system with nanofinger tetramers under a monolayer WSe_2_, in which the red and blue spheres represent for the in-plane excitons. (c) Strong spilling-out plasmonic electric field in free space generated by the ultrathin ta-C film coated nanofingers. (d) Strong interaction between the coupled plasmons and in-plane excitons.

In this letter, we demonstrated the coupling of plasmon–excitons by placing the monolayer WSe_2_ on the collapsed gold nanofingers with sub-nanometer gap scale, in which the vibration direction of excitons in monolayer WSe_2_ and extremely strong in-plane plasmonic electric field are completely parallel. The light emitting properties of polaritons driven by plasmonic nanofingers are studied. As shown in Figure 1a, the collapsed Au nanofingers were fabricated via the collapsible Au nanofingers by precoating a 1 nm tetrahedral amorphous carbon (ta-C) film on them, in which 2 nm ta-C dielectric gap was formed by twice the ta-C film thickness. This structure enables the precise tailoring of the gap plasmon modes to realize an in-plane electromagnetic field with huge enhancement and nanoscale mode volume. After transferring of a monolayer WSe_2_ upon the collapsed Au nanofingers tetramer arrays, completely parallel in-plane plasmons and excitons in WSe_2_ will induce a strong coupling in which the coupling strength reaches up to 68.5 meV at room-temperature, as shown in Figure 1b and c. Interestingly, it is found that exciton–polaritons show completely different light emitting properties by compared to that for monolayer WSe_2_ on silicon or on other plasmonic nanostructures [13, 17]. The results show that photoluminescence (PL) spectra of WSe_2_ driven by plasmonic nanofingers can avoid the modulation by temperature from 300 K down to 77 K. According to the theoretical analysis through the first-principle calculation, these plasmonic nanofingers can produce an extremely strong in-plane electric field as shown in Figure 1d, which effectively increase the dielectric screening of the system. The strong screening effects will modulate the band structure of WSe_2_, leading to a lower polariton energy and influencing the temperature dependence on PL. Our finding provides a novel way for light emission manipulating of excitons, leading to a significant application potential in on-chip plasmonic polaritonic devices.

## 2 Results and discussion

**Figure 2:**
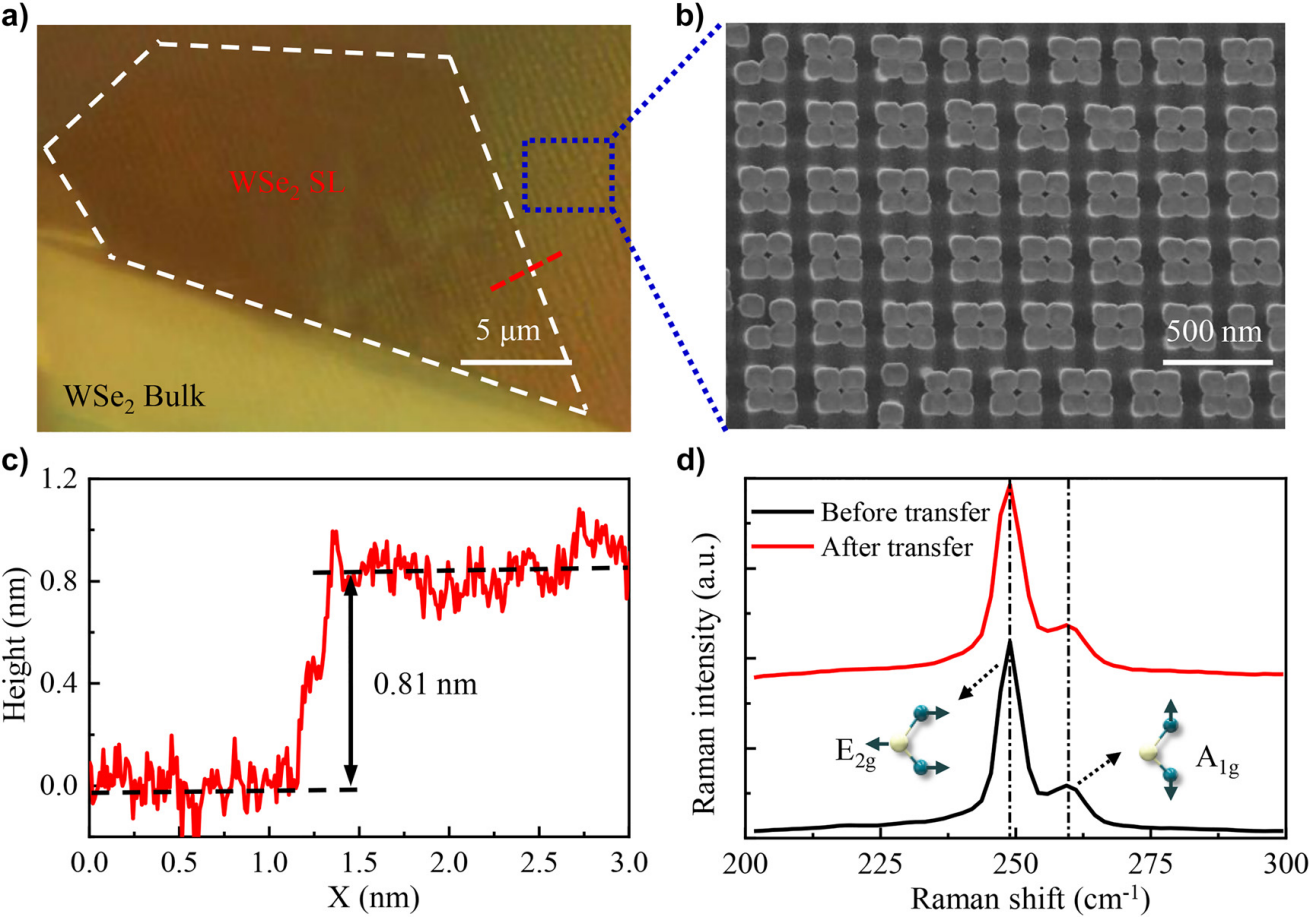
Monolayer WSe_2_ transferred onto plasmonic nanofinger arrays. (a) The microscope image of the as-prepared WSe_2_-nanofinger sample. (b) Top-view SEM image of 1 nm ta-C coated Au nanofingers after collapse at the blue rectangle position in (a). (c) Height profile scanned with an AFM along red dash-line of sample in (a). (d) Raman spectra of monolayer WSe_2_ before and after transfer from silicon on insulator (SOI) to nanofingers.

Figure 2a shows an optical microscope image of monolayer WSe_2_ on the top of collapsed nanofingers. Herein, the flexible nanofingers are firstly fabricated by our well-developed nanoimprint lithography (NIL) and reactive-ion etching [26]. The typical diameter of each nanofinger is 70 nm and its height is finely controlled to 300 nm including UV nanoimprint resist fingers with 50 nm Au layer on top for forming tetramer structures in the collapsing process. In addition, the nanofingers can be well defined with different geometric parameters by the initial lithography mold and reproduced precisely by NIL. Subsequently, a 1 nm ta-C film was deposited on the top of Au nanofingers by filtered cathodic vacuum arc (FCVA) technology, where the samples were tilted at 45-degree angle and rotated for a better coverage on the cylinder wall of each nanofinger. The refractive index of the 1 nm ta-C is measured to 2.44, which is important for producing strong free space spilling-out of the enhanced electromagnetic field [27]. In addition, low electron affinity (1.5 eV) of the ta-C generates the high tunneling barrier height (5.1–1.5 = 3.6 eV) for this gap plasmon system, so that the coupled electric field in the gap can avoid the attenuation caused by quantum tunneling effects. Finally, after soaked into ethanol and air-dried, the adjacent nanofingers are physically contacted to form tetramer via a capillary force-induced collapsing process, in which the gap size is defined by twice of the ta-C thickness. The details of the nanofingers can be found in our previously report [27]. The top view of scanning electron microscopy (SEM) image with a scale bar of 5 μm for nanofinger tetramers are shown in Figure 2b. In this work, the monolayer WSe_2_ samples were firstly mechanically exfoliated onto the substrate, which was verified by atomic force microscope (AFM) measurement, as shown in Figure 2c. In addition, as layered WSe_2_ is lowered from bulk to monolayer, its typical Raman peak centered at ∼250 cm^−1^ will split into two peaks at ∼250 cm^−1^ and 261 cm^−1^, respectively, which are assigned to the in-plane (E_2g_) and out-of-plane (A_1g_) modes [28], as shown in Figure 2d black line. After transferring the monolayer WSe_2_ on the top of collapsed nanofingers, the Raman results in Figure 2d red line show two unchanged Raman peak of E_2g_ and A_1g_ modes, which means that the strain effect can be negligible here.

**Figure 3:**
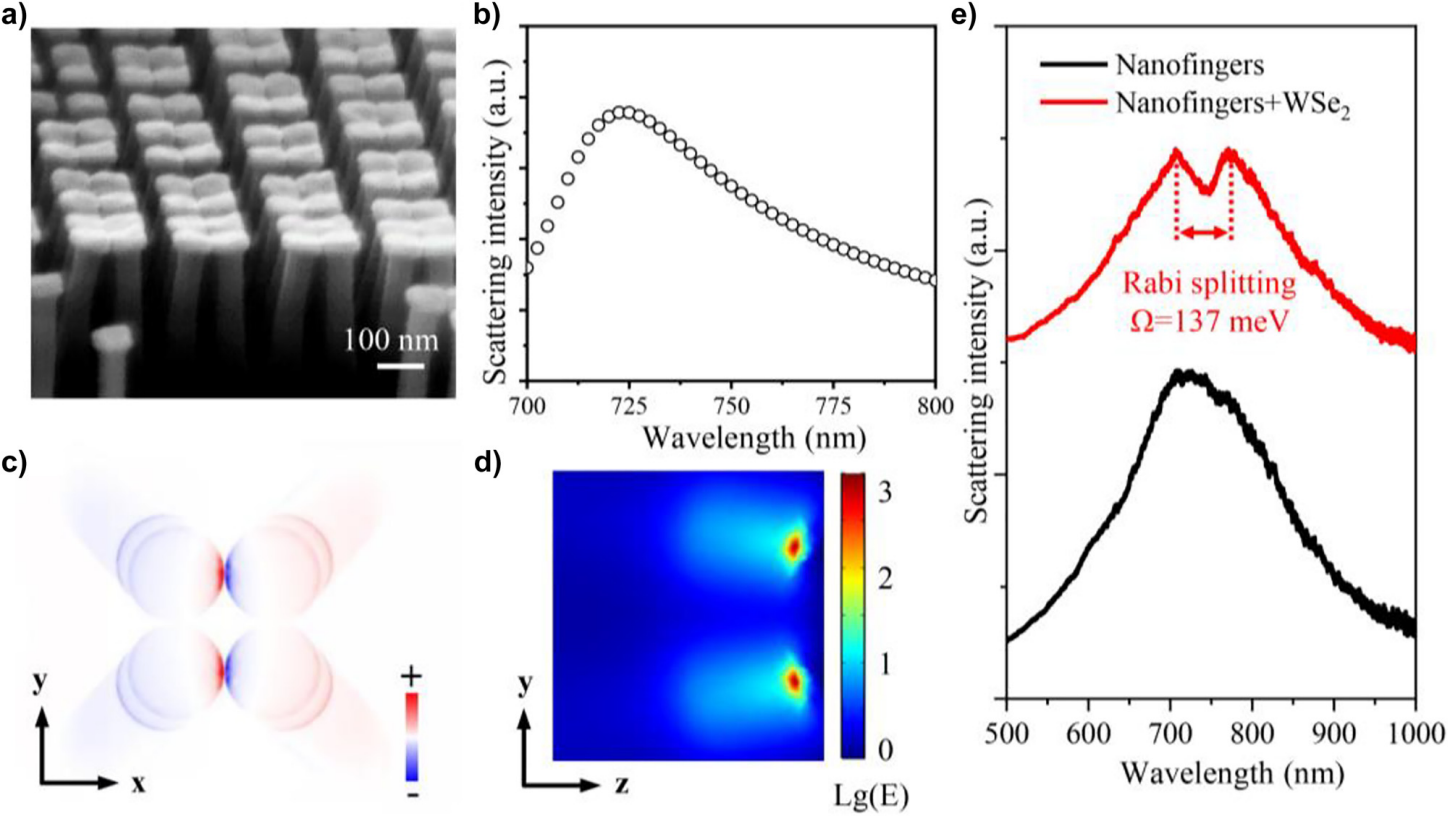
Structural and spectral characterization for plasmonic nanofingers. (a) Oblique-view SEM image of 1 nm ta-C coated Au nanofingers after collapse. (b) Numerical simulation of the scattering spectra for the collapsed nanofingers. (c) and (d) Charge distribution (in *x*–*y* plane) and electric field distribution (in *y*–*z* plane at the gap area) for the nanofinger tetramer at scattering peak of 723 nm. (e) The measured dark field scattering spectra for the collapsed nanofingers tetramer with (red curve) and without (black curve) WSe_2_.

In order to demonstrate the localized electric field enhancement generated in our proposed collapsed nanofingers as shown in Figure 3a, the simulations based on the commercial software (COMSOL Multiphysics) are performed. The parameters of the nanofingers in the simulations are consistent to the experimental observation. The results show that there is a dominant scattering peak at 723 nm with an incident electric field polarized along *x* direction, which corresponds to a bonding-dipole plasmon resonance mode, as shown in Figure 3b. The typical surface charge distribution in Figure 3c confirms this resonance mode. Furthermore, at the 723 nm excitation, the calculated electric field enhancement in *y-z* plane at the gap region can reach up to 1000 times with nanometer-scale mode volume as shown in Figure 3d. Due to the modification of ta-C film to the collapsed Au nanofingers, the coupled in-plane strong electric field can spill out from the dielectric gap, which is beneficial for the strong coupling between the plasmon and the excitons [29]. In order to demonstrate these spectral properties experimentally, dark field scattering measurements were performed, as shown the black line in Figure 3e. The results show that the experimentally dark field scattering spectra can fit the numerical simulation except some slight differences in resonance wavelength. This could be attributed to the geometry deviation in the experiments. As shown the red line in Figure 3e, after transferring a monolayer WSe_2_ to the top of collapsed nanofingers, an obvious dip at 745 nm wavelength appeared which is close to the peak of neutral exciton in monolayer WSe_2_. And the scattering spectral show two peaks at 707 nm and 772 nm wavelength instead of only one peak like in the case without WSe_2_. Compare with the physical phenomenon described in the reference [13], a strong coupling between plasmon and excitons are observed here, in which a vacuum Rabi splitting is obtained at zero detuning.

**Figure 4:**
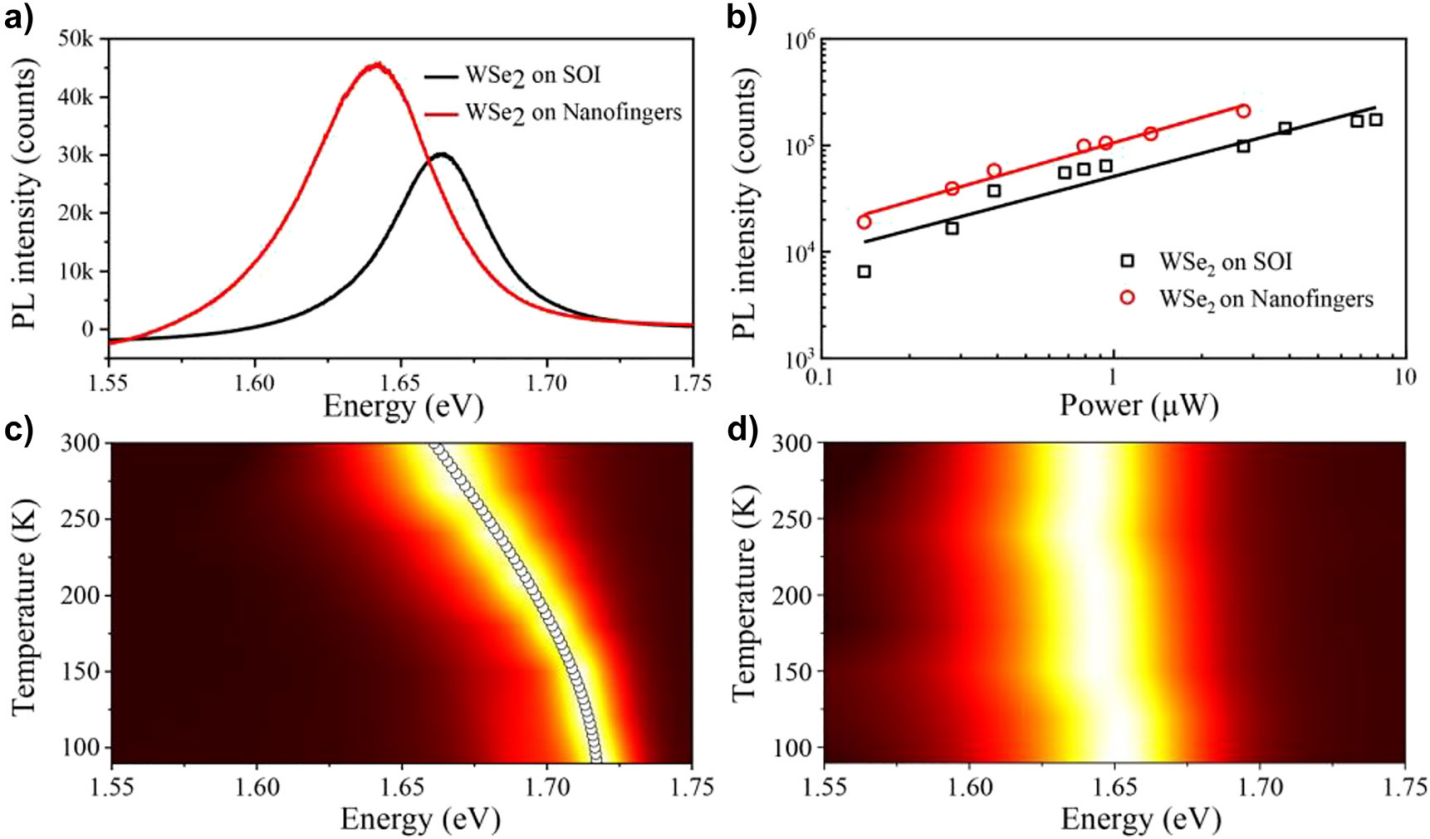
Characterizations of photoluminescence for WSe_2_ on SOI and nanofingers. (a) PL spectra of monolayer WSe_2_ on SOI (black) and nanofingers (red) at room temperature. (b) PL peak intensity of WSe_2_ on SOI (black) and nanofingers (red) with different incident laser power. (c) The measured temperature dependent PL spectra of WSe_2_ on SOI (the theoretical fitting of PL peak marked by hollow circle line). (d) Temperature dependent PL spectra of WSe_2_ on nanofingers.

The plasmon–exciton coupling can be described by the quantum mechanical Jaynes–Cummings model. For simplicity, the collective response of the excitons can be treated as a “super oscillator”, and the system can be qualitatively described as two coupled oscillators [30]:

$$\left(\begin{array}{ll} E_{\text{pl}}-i\Gamma_{\text{pl}}/2 & g\\ g & E_{0}-i\Gamma_{0}/2 \end{array}\right)\left(\begin{array}{l} \alpha \\ \beta \end{array}\right)=E\left(\begin{array}{l} \alpha \\ \beta \end{array}\right)$$

where *E*_pl_ and *E*_0_ are the energy of nanofinger tetramer plasmons and the WSe_2_ excitons, *g* is the coupling strength, Γ_pl_ and Γ_0_ represent the dissipation rates, *E*, *α* and *β* stand for the energy and eigenvector components of the quasi-particles. Here, we assume that the widths of excitons and plasmons are very small compared to their energies, then we can get the solution $E_{\pm }=\frac{1}{2}\left(E_{0}+E_{\text{pl}}\right)\pm \sqrt{g^{2}+\frac{1}{4}\delta^{2}}$ , where *δ* = *E*_pl_ − *E*_0_ is the detuning. By fitting the dark field scattering spectra in Figure 3e to the coupled oscillators model, the energy of the upper polariton branches and lower polariton branches are 1.754 eV and 1.606 eV, respectively. *E*_pl_ = 1.720 eV which is determined from the black line in Figure 3e, and *E*_0_ = 1.664 eV which is determined from the PL spectra of monolayer WSe_2_ as shown in black line in Figure 4a. Thus, the coupling strength *g* is 68.5 meV, which means a vacuum Rabi splitting of $\Omega =2g{|}_{\delta =0}=137\text{ meV}$ can be obtained at room-temperature.

In order to explore the effect of extremely localized electromagnetic field enhancement on the in-plane excitons of monolayer WSe_2_, the PL spectra of monolayer WSe_2_ on our proposed nanofingers are measured and SOI substrate as the reference. The results show that the PL spectra for the WSe_2_ on collapsed nanofingers indicate an enhancement compared to WSe_2_ on SOI with the increasing incident laser power, as shown in Figure 4a and b. According to the linear fitting curve of power, the PL peaks have all been attributed to the excitons rather than biexcitons. More interestingly, the PL peak of polariton shows a red shift and the PL linewidth is slightly broadened at room-temperature, which has not reported previously according to our knowledge. In order to reveal the mechanisms underlying the phenomenon, we further measured the PL of WSe_2_ on collapsed nanofingers and SOI from 300 K (room temperature) down to 77 K, as shown in Figure 4c and d. The PL of the monolayer WSe_2_ on our proposed plasmonic nanofingers show obviously different dependences on temperature compared to that on SOI. The PL peak of WSe_2_ on SOI shows a red shift and the PL linewidth is broadened with increasing ambient temperatures which is consistent with the previous reports [31]. However, both the PL peak and linewidth still are nearly unchanged for the WSe_2_ on the collapsed nanofingers, which means the luminescence properties of excitons are modulated through this strongly localized electromagnetic field.

**Figure 5:**
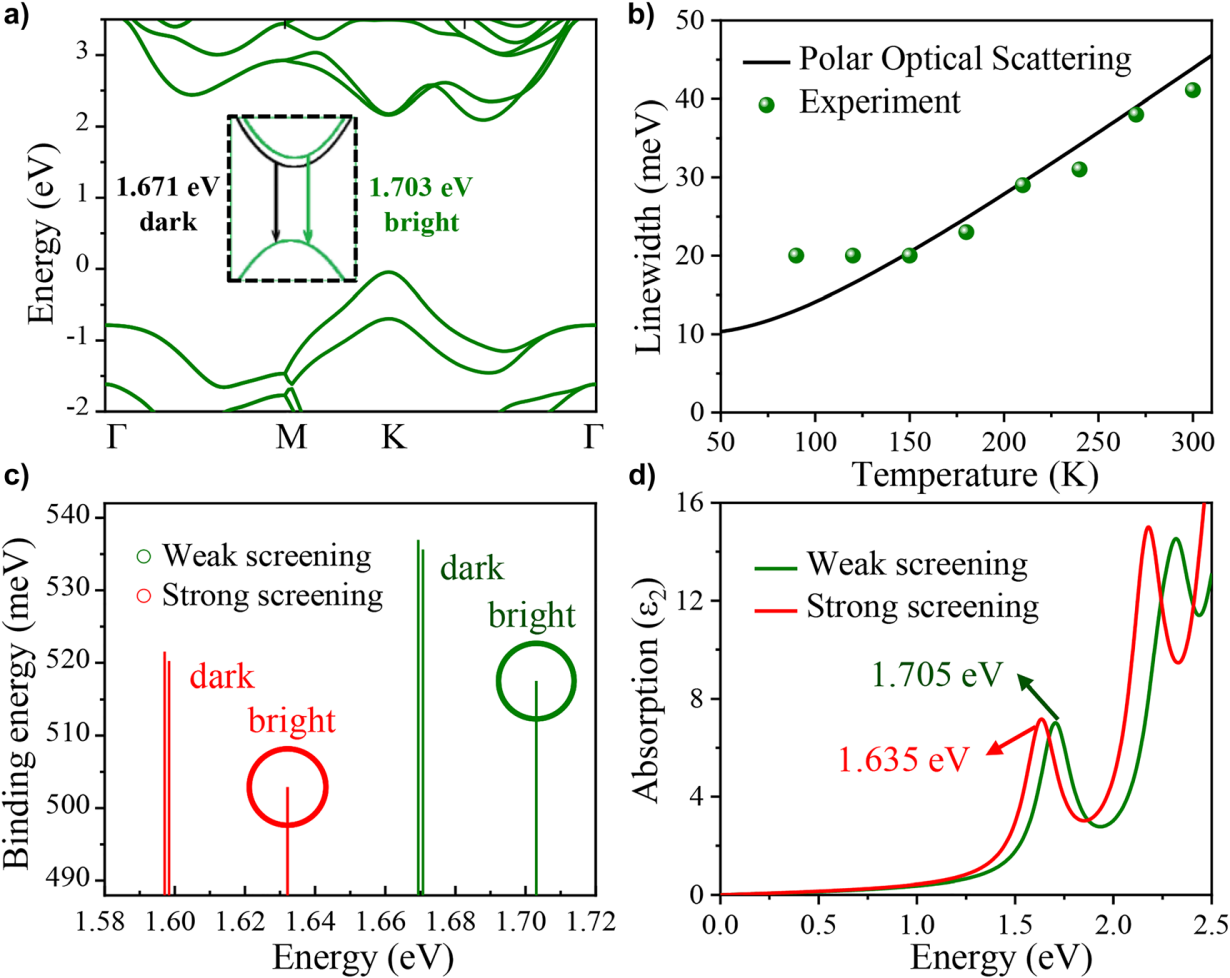
Theoretical calculation of WSe_2_ on SOI or nanofingers. (a) Electronic structures of monolayer WSe_2_ on SOI, Inset: enlarged view of electron transition from the bottom of conduction band to the top of valence band. (b) PL linewidth as a function of temperature. (c) Dark and bright excitons and their corresponding binding energy under weak and strong screening. (d) Imaginary part of dielectric function under weak and strong screening.

We carried out a theoretical analysis to further study the physical mechanisms underlying the observed phenomena. The optical properties of monolayer WSe_2_ are dominated by electron–hole pairs bound by Coulomb interactions, which are called the excitons. Figure 5a demonstrates an electronic band structure of monolayer WSe_2_, where a direct bandgap is indicated at *K* point. It is noted that the excitons could be either dark or bright. For the bright excitons, the electrons and holes have antiparallel spins and could recombine through photon emission, whereas dark excitons have parallel spins, and direct emission of photons is not allowed for spin momentum conservation. The dark excitons have lower energy (i.e. 1.671 eV) than the bright excitons (i.e. 1.703 eV) due to spin-split states, which is caused by the inversion and rotational symmetry breaking as well as strong spin–orbit coupling. The first bright exciton energy is within 0.9% of the measured low-temperature PL peak. According to the theoretical calculation of pure semiconductor materials in reference [32], the temperature dependence of the PL peak can be fitted by a Bose–Einstein oscillator model:

$$E\left(T\right)=A_{0}+\frac{A_{1}}{{\text{e}}^{\omega_{0}/k_{\text{B}}T}-1}$$

where *T* is the temperature, *k*_B_ is the Boltzmann constant, and *A*_0_, *A*_1_, *ω*_0_ are the fitting parameters. The model fits well with experimental data with fitting parameters *A*_0_ = 1.718 eV, *A*_1_ = −0.243 eV, and *ω*_0_ = 42.85 meV, as shown the white dotted line in Figure 4c. The linewidth of the measured PL spectra is nearly constant at temperatures below 150 K, and increases linearly at higher temperatures, which shows a similar trend to polar optical phonon scattering and can be calculated by [33, 34]:

$$\Gamma^{\text{LO}}\left(T\right)=\frac{e\omega \left(\frac{1}{\epsilon_{\infty }}-\frac{1}{\epsilon_{0}}\right)}{4\pi \sqrt{\frac{2E_{k}}{m^{*}}}}\left[\left(n_{q}\sinh^{-1}\sqrt{\frac{E_{k}}{h\omega }}\right)+\left(n_{q}+1\right)\left(\sinh^{-1}\sqrt{\frac{E_{k}}{h\omega }-1}\right)\right]$$

where *e* is the elementary charge, *ω* is the phonon frequency, *ε*_∞_ and *ε*_0_ are the high frequency and static dielectric constants, *E*_*k*_ is the exciton energy, *m* ^∗^ is the exciton mass, and *n*_*q*_ is the phonon distribution function, respectively. For simplicity, we use the highest longitudinal optical (LO) phonon frequency (238.3 cm^−1^) as *ω*. The black line in Figure 5b shows the calculated linewidth contributed by the polar optical scattering, confirming its dominant contribution. The slight discrepancy is due to other scatterings such as phonon-mediated exciton scattering between the bright and dark states [35].

The density of free electrons is relatively high due to the nanofinger structure, increasing the screening of the system. Here, the electron–electron interactions can be separated into two parts of short-range and long-range. The strong screening leads to a much shorter screening length, where the long-range Coulomb interactions become negligible. By applying a screened Coulomb potential to the exchange interaction, the HSE06 and HSE03 functions can mimic weak and strong screening effects, respectively [36, 37]. As shown in Figure 5c, the energy of the first bright excitons under strong screening have a much lower energy (1.632 eV) than that under weak screening (1.705 eV). Therefore, the absorption peak in Figure 5d is redshifted from 1.705 eV to 1.635 eV, which is well consistent with the measured PL peak in Figure 4.

Now, we explain how screening influences the temperature dependence of the PL spectra. As shown before, the temperature dependence is dominated by polar optical phonon scattering. The LO modes have an associated electric polarization wave, causing a long-range electric field that scatters the excitons. However, the electric field generated by the LO phonons tends to be screened by the free electrons in the presence of nanofingers, which reduces the strength of exciton–LO phonon Fröhlich interaction. Thus, a much weaker exciton–phonon interaction is expected, suppressing the temperature dependence of the PL peak. As a result, the exciton scattering becomes dominant due to the interaction in the electronic system. The linewidth can be calculated from the imaginary part of the self-energy of the electron–hole pairs [38, 39]:

Γe−h=Im∑e−h2=12∑e,h,k|Ake,h|2(Im∑e+Im∑h)

where ∑^e−h^, ∑^h^, and ∑^e^ is the exciton, hole and electron self-energies, respectively, and *A*_k_^e,h^ is the electron–hole amplitude. The calculated linewidth of the electronic system is 51.45 meV, which agrees well with the linewidth of the measured PL peak in the presence of nanofingers.

## 3 Conclusions

In summary, we demonstrated the possibility of studying in-plane exciton of monolayer TMD materials in a form of exciton–plasmon polariton by utilizing the well-designed plasmonic nanofinger tetramers which provide the extremely localized enhanced electric field. After transferring a monolayer WSe_2_ onto the top of plasmonic nanofinger tetramers array, a strong in-plane plasmons–excitons coupling with coupling strength up to 68.5 meV at room-temperature were observed. Comparing with the case of monolayer WSe_2_ on SOI, the room-temperature PL spectrum for plasmon-coupled monolayer WSe_2_ showed a spectral red shift with slight linewidth broadening. Furthermore, we experimentally observed that the luminescence of exciton–plasmon polariton is inert to the temperature probably due to the dielectric screening effect originated from the high density of free electrons oscillation of nanofinger tetramers driven by plasmon field, which could be elucidated by theoretical analysis based on first-principle calculation. Our work provides a novel route for luminescence modulation of exciton in 2D material, which has a significant application potential in on-chip plasmonic polaritonic devices.

## 4 Methods

### 4.1 Sample preparation

The flexible polydimethylsiloxane (PDMS) nanofinger arrays were fabricated by our well-developed nanoimprint lithography and reactive-ion etching. The typical diameter and height of each nanofinger is 70 nm and 300 nm, respectively. The periodicity in the tetramer arrays is 500 nm. Then, Au layers with thickness of 50 nm were deposited onto the top of nanofingers by e-beam evaporation. An ultrathin ta-C film with thickness of 1 nm was covered on the top of Au nanofingers by filtered cathodic vacuum arc (FCVA) technology, where the samples were tilted at 45-degree angle and rotated for a better coverage on the cylinder wall of each nanofinger. Finally, after soaked into ethanol and air-dried, the collapsed nanofingers done. The monolayer WSe_2_ samples were mechanically exfoliated from bulk materials and then transfer on the top of collapsed nanofingers by wetting process.

### 4.2 Electromagnetic simulation

The numerical simulations of the scattering spectra for the Au nanofinger tetramer coated with ta-C layer were based on the commercial software (COMSOL Multiphysics). The geometrical parameters of the nanofingers in the simulations are consistent to the ones in the experiment. And the gap size at the top (or bottom) of the adjacent nanofinger is zero (or 130 nm). The refractive index of PDMS and ta-C are set as 1.41 and 2.44, respectively. The permittivity of Au is from COMSOL built-in material library. A plane wave irradiates normally along the negative *z*-axis direction with *x*-direction polarization, and the collapsed nanofinger tetramer is surrounded by a perfect matched layer condition.

### 4.3 Spectral measurement

The Raman spectra were recorded using a Renishaw inVia Raman microscope at excitation wavelength of 514 nm. The excitation wavelength of self-build dark field scattering system and PL system were 400–1000 nm and 532 nm, respectively. The temperature of sample table was determined by the injection rate of liquid nitrogen. A 50× standard objective lens was used in all spectral measurement system.

### 4.4 Theoretical calculation

Based on density functional theory (DFT), first-principles calculations were performed using the Vienna ab-initio simulation package (VASP) [40]. The projector-augmented-wave potential was used with W 5p^6^5d^4^6s^2^ and Se 4s^2^4p^4^ valence states. Based on convergence tests, a kinetic energy cut-off set to 300 eV and a Γ-centred 12 × 12 × 1 *k*-mesh to sample the electronic Brillouin zone were used. The convergence parameters for structural relaxations included an energy difference within 10^−6^ eV and a Hellman–Feynman force within 10^−4^ eV/Å. The energy cut-off for the response function was set to be 200 eV. A total of 48 (valence and conduction) bands were used, and the convergence of our calculations had been checked carefully. The 12 highest valence bands and the 12 lowest conduction bands were included as basis for the excitonic state to compute the dielectric function.
